# Improving the energy balance between microwave pretreatment and anaerobic digestion with centrifuged biosludge

**DOI:** 10.1186/s40643-025-00903-7

**Published:** 2025-10-02

**Authors:** Sukru Aslan, Ali Alhraishawi

**Affiliations:** 1 Department of Environmental Engineering, Sivas Cumhuriyet University, Engineering Faculty, Sivas, Türkiye; 2 Sivas Cumhuriyet University Graduate School of Natural and Applied Sciences, Sivas, Türkiye; 3 Department of Chemical Engineering, Engineering Faculty, Misan University, Amara, Iraq

**Keywords:** Anaerobic digestion, Biosludge, Energy assessment, Microwave, Pretreatment

## Abstract

The effect of pretreatment of raw biosludge (Bs) and centrifuged Bs (CBs) with microwave (MW) operated at constant conditions before an anaerobic digestion (AD) process on the energy requirement and production of hybrid system was investigated. With the successful breakdown of Bs and CBs by the MW irradiation, the soluble COD concentrations increased from average value of about 76 mg/L to 300 mg/L and 6400 mg/L for the Bs and CBs, respectively. After the MW disintegration, the protein and sugar concentrations were about 10 and 22 times higher in the pretreated samples compared to the biosludges. While, the total and volatile solids (TS and VS) concentrations in the liquor declined. The increase of readily bioavailable organics in pretreated samples improved about 22% CH_4_ production in AD units, compared to the raw Bs and CBs. It was determined that the energy requirement of MW irradiation decreased about 3 times with the centrifugation of Bs. Although the applied whole models were well correlation, the kinetic analysis showed that Transference Function was the best model with the highest correlation coefficient (R^2^ between 0.953 and 0.998) for predicting the most accurately CH_4_ production. The nutrients concentrations in effluent of AD process was significantly higher than the liquors obtained after the MW irradiation was observed.

**Graphical abstract:**

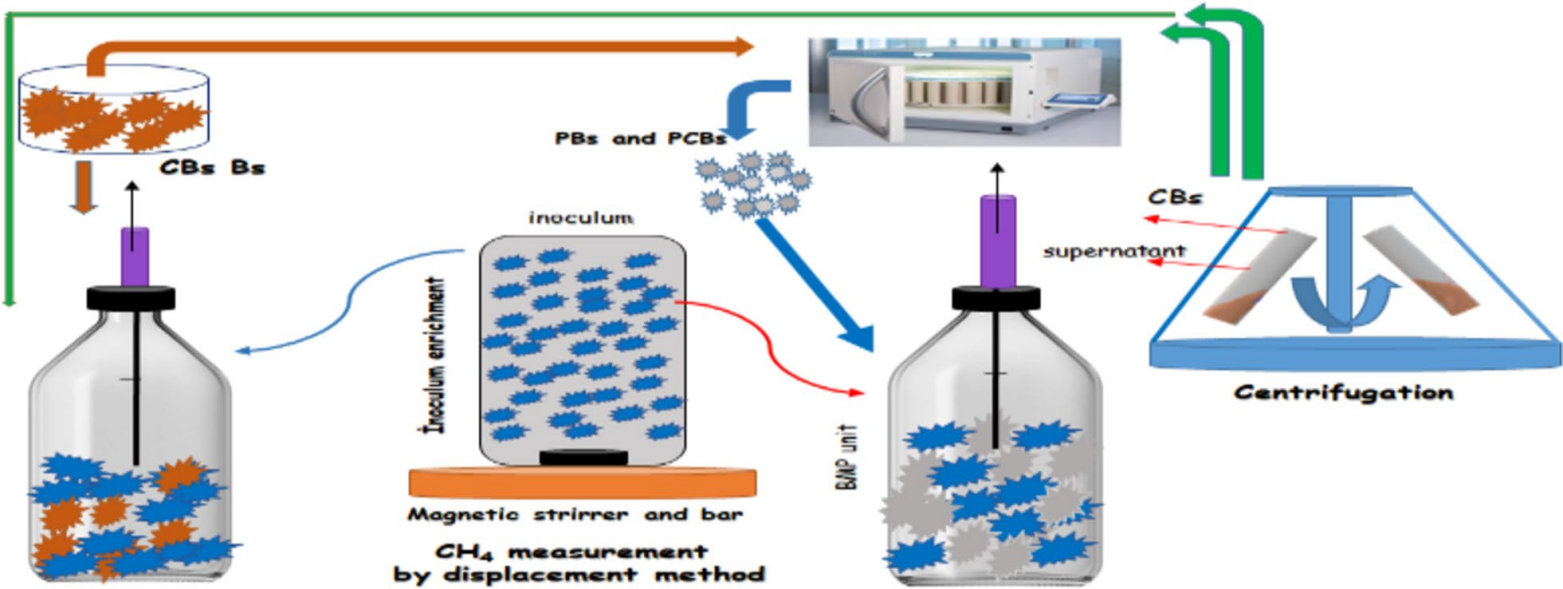

## Introduction

An industrial and domestic wastewater must be treated to maintain the ecological balance before being discharged into the environment. In general, the biological treatment process is applied in a sequentially consisting of anaerobic, aerobic and anoxic stages to remove organic matter, ammonium and phosphorus from municipal wastewater. The main goal in biological treatment is to convert dissolved and particulate pollutants into biomass, which is an easily settleable form, and end products such as CO_2_, H_2_O, N_2_, CH_4_, etc., depending on the applied process. In addition to the pollutants in wastewaters utilized as substrates by biomass, some more complex organic pollutants such as pharmaceuticals, surfactants, polyaromatic hydrocarbons, etc., and significant portion of heavy metals are sorbed or accumulated to the Bs (Christodoulou and Stamatelatou 2016). The complex component of Bs is made up of ~ 3.5% DNA (values as % volatile solids, VS), proteins (25% ~ 62%), carbohydrates (7% ~ 19%), humic compounds (8% ~ 29%), and bacterial biomass (10% ~ 24%) (Jiang et al. 2021).

The disposal of biosludge (Bs) containing low concentration of dry solids produced in the biological wastewater treatment plant (WWTP) is one of the most important environmental problem. Prior to the disposal of Bs, it must be treated due to the environmentally harmful components and high volume. Among the methods, an anaerobic digestion (AD) process is considered as an economical option for the Bs treatment, which contain high amount of organic compounds, because of the volume reduction and CH_4_ production. It was reported that the conversion rates of proteins, lipids, and carbohydrates to CH_4_ in the AD reactor were theoretically 1014 NL CH_4_/kg VS, 740 NL CH_4_/kg VS, and 370 NL CH_4_/kg VS, respectively (Jiang et al. 2021). Náthia–Neves et al. (2018) reported that the produced fraction of biogas varies 50–75% CH_4_, 25–45% CO_2_, and trace volume of 2–7% H_2_, H_2_S, and N_2_ by the utilization of organic compounds in AD process.

The AD process consists of mainly three stages: hydrolysis, acid formation and CH_4_ production, which are carried out by special bacteria. The hydrolysis, where high molecular weight organic compounds are converted to low molecular, is considered as the rate − limiting step. Nevertheless, the prolonged hydraulic retention time (HRT) and insufficient substrate digestion for the Bs treatment in AD process are considered as the main issue. The operational conditions of AD reactor such as temperature, pH, moisture, C/N ratio, organic loading rate, and HRT effect not only the microbial communities but also CH_4_ production efficiency (Náthia − Neves et al. 2018).

The yield of AD process depends on the hydrolyzes of particulate organics in Bs. Bioenergy in the AD process can be improved by the conversion of hard − to − degrade organic molecules (in the polymeric network and within the cell) with the pretreatment into easily consumable organics for bacteria (Kor − Bicakci et al. 2019). Pretreatment, which reduce or eliminate the hydrolysis period in the AD process, is considered as a suitable method to destroy the Bs wall and to release substrates into the liquid medium that can be consumed more easily by bacteria. Over the past 30 years, various Bs pretreatment techniques including mechanical, chemical, and thermal have been investigated (Zhang et al. 2017).

Nonetheless, several researches assessing the use of microwave (MW) heating in Bs hydrolysis have been conducted throughout the past ten years (Tyagi and Lo 2013). Rapid heating, pathogen reduction, controllability, compactness and low total cost are cited as benefits of MW (Park et al. 2010). Kor − Bicakci et al. (2019) reported that the MW pretreatment provided noticeably more cellular disintegration of about four to five times compared to raw Bs. Yigit and Onur (2020) reported that the MW pretreatment provided the acceleration of hydrolysis step in AD reactor and also not only higher biogas production and but also better biogas quality in which CH_4_ content was higher. However, because the recalcitrant insoluble fibrous components of macroalgae (*Laminaria* spp. and *Sargassum* spp.), the reduction of CH_4_ yield in AD process after the MW pretreatment was reported (Paletta et al. 2025).

The parameters such as target temperature, heating rate, duration, and the solid content of Bs influence the MW irradiation effectiveness (Aslan et al. 2024). Operating the MW at low heating rates (1 − 4 °C/min) were reported to be more efficient in the release of organic matters from the Bs in the form of sugars and proteins, which improved CH_4_ generation in the AD process (Koupaie and Eskicioglu 2016). Chan et al. (2007) found that the organic compounds became more soluble at the MW target temperature between 80 °C and 120 °C and result in the enhanced CH_4_ generation was obtained. Although the Bs could be well degradable anaerobically, the increase in CH_4_ production in AD process after the disintegration might not be met the energy requirement of MW operation (Alhraishawi et al. 2024 and 2025). Because of the Bs water content, which is about % 97 − 98 produced in the suspended growth process, an energy requirement of MW irradiation is high. With the high water content of Bs absorb more MW irradiation energy result in a relatively low temperature increase due to its high thermal capacity (Alhraishawi et al. 2024).

The disintegration of Bs with MW energy requirements depends heavily on the characteristics of Bs, which concentration, composition, and physical properties. Generally, for the MW pretreatment of Bs contained high concentrations of TS require less energy compared to lower. Cano et al. (2015) reported that 0.2 kWh of more energy was required in the MW process for each additional kilogram of TS in Bs.

Increase in the Bs temperature during MW irradiation is directly related to the content of water with dipolar molecular structure absorbs energy and gradually increases its temperature (Tang et al. 2010). Previous experimental studies indicated that the MW process was not economic due to its high energy input for the pretreatment of raw Bs contains low amount of TS (Kor − Bicakci et al. 2019). Our previous studies also indicated that the hybrid MW irradiation and AD process for the raw Bs were not satisfied the economically feasible considering only for the operational conditions (Alhraishawi et al. 2024 and 2025). The TS/VS concentration of Bs plays a crucial role in assessing the energy and economic feasibility of pretreatment processes.

In this study, the effect of biosludge TS/VS content on MW energy requirement and CH_4_ yield in the anaerobic digestion process for the raw Bs and centrifuged Bs contained 3.4% TS was investigated. To evaluate the Bs degradation efficiency, the changes of nutrients and organic components in the MW pretreatment and AD process were determined. Additionally, the experimental data of studied substrates in AD process were applied to various kinetic models, the ability of kinetic models results to represent the experimental data was determined and the model parameters were calculated.

## Materials and methods

### Bs collection

The Bs used in study was taken from the recycling line of municipal biological WWTP in Sivas/Türkiye. The plant was constructed as an extended aeration activated sludge process for treating the organics and nutrients (ammonium/nitrate and phosphorus). After collecting the Bs, the sample was immediately transported to the laboratory (Environmental Engineering Laboratory/Sivas Cumhuriyet University), and analyses were conducted and experiments were performed with the MW for centrifuged and raw samples on the same day to avoid fermentation or other microbial activities. After disintegration of the samples with MW irradiation centrifuged/filtered were stored in a refrigerator at + 4 °C.

### MW irradiation

The Bs disintegration was carried out with a closed − vessel MW accelerated reaction system (MA179 − 001; MAXI − 44; Milestone). The MW device could be operated at a frequency and powers of 2450 MHz and 900W/1800 W, respectively. The MW had a maximum temperature of 200 °C and a pressure of 35 bar. The MW had a rotating carousel with 44 sealed containers of 100 mL capacity each. The device had fiber optic probes to control operating temperature and pressure during the heating. Operating conditions of MW was adjusted to the ramping rate and target temperature of 2 °C/min and 90 °C, respectively. The input power of 900 W was applied and the MW irradiation was continued 5 min at the target temperature. Operating conditions were selected based on our previous studies (Alhraishawi et al. 2024; Aslan et al. 2024).

### Centrifuge Bs preparation

The Bs was centrifuged and the most of supernatant volume was drawn. Remaining liquid at the upper layer of CBs was intentionally kept to avoid high TS concentrations that could negatively affect the heat transfer and absorption of MW energy in the containers (Fang et al. 2017). After the centrifugation, the TS content increased from about 1.27% to 3.4%.

### Enrichment of anaerobic bacteria for BMP test

The Bs obtained from Sivas WWTP was placed into 5L batch reactor for the enrichment of anaerobic organisms for BMP tests. In order to provide suspended growth condition, the reactor was mixed with a magnetic stirrer and the temperature was adjusted to 35 ± 2 °C. The initial pH of mixed liquor set to 7.5 ± 0.5 by using NaOH and H_2_SO_4_ solutions. Concentration of O_2_ in the reactor was kept constant lower than 0.5 mg/L by adding appropriate amount of Na_2_SO_3_. Because the anaerobic organisms had a low growth rate, the batch reactor was operated at the sludge retention time of 40 days. For the SRT, required volume of mixed liquor was withdrawn daily from the reactor and sCOD analysis was performed on the samples to monitor biological activity. The pH and O_2_ concentration of reactor were also measured. Enrichment of bacteria was lasted 6 months with daily Bs additions.

### Biochemical methane potential test

The BMP tests were carried out by using Bs, pretreated biosludge (PBs), CBs, and pretreated centrifuged Bs (PCBs). The BMP tests were conducted at a ratio of 1/1 (volume/volume) with the samples and inoculum organisms enriched in the laboratory. The sample and inoculum organisms, which were the total volume of 200 mL, were poured into each 500 mL serum bottles. Before the BMP experiment, to provide an anaerobic condition, O_2_ was removed by passing N_2_ gas for about 5 min through the liquid. In order to prevent O_2_ entrance into the bottles, screw caps and rubber septa stoppers were used. After the bottles were covered with an aluminum foil to avoid light exposure, they were placed in a water bath shaker (Julabo SW23), which was operated at the temperature and rotation speed of 37 °C and 120 rpm, respectively. CH_4_ production in the units was determined by gas − liquid displacement method. Daily CH_4_ volume was measured until the production was no longer detectable. In order to ensure reliability of the CH_4_ measurements, three BMP tests were performed for each sample.

### Kinetic studies

Anaerobic degradation potential in the BMP units were evaluated by fitting the cumulative CH_4_ production data with four nonlinear models; First − order kinetic, modified Gompertz, Logistic and Transference Function. The models formulas are presented at Eq. 1, 2, 3, 4 (Alhraishawi et al. 2024, 2025).

1 $$A={A}_{0}\times \left\{1-exp\left(-k.t\right)\right\}$$

2 $$A={A}_{0}\times \left\{1-\right.\left.exp\left[-{\mu }_{m}\times \left(t-\right.\right. \lambda )/\left.{A}_{o}\right]\right\}$$

3 $$A= {A}_{0}\times exp\left\{-exp\left.\left[\frac{{\mu }_{m}\times e}{{A}_{0}}\times \left(\uplambda -t\right)+1\right]\right\}\right.$$

4 $$A=\frac{{A}_{0}}{\left[1+exp\left\{\left\{4\times {\mu }_{m}\times \frac{\uplambda -t}{{A}_{0}}\right.+2\right\}\right]}$$

where A: Cumulative CH_4_ production at time t (mL/g VS); A_0_: Maximum CH_4_ potential yield (mL/g VS); λ: Lag phase (days)–the time before significant CH_4_ production begins; k: Hydrolysis rate constant (1/day)—related to the breakdown of organic compounds; μ_m_: Maximum CH_4_ production rate (mL/g VS.day)—the highest rate at which CH_4_ was produced; e: Euler’s number (2.7183), a constant used in exponential functions.

At the first − order kinetic model, it is assumed that the substrate availability is limiting factor and hydrolysis governs the overall process, while the conditions necessary for maximum biological activity, lag phase, and system failures are not predicted. An empirical non − linear regression model, modified Gompertz is applied to the experimental data in order to describe the exponential growth rates and lag phase duration during the AD process. By the Transfer function model, the maximum CH_4_ production is predicted based on accumulated CH_4_ production over time in the digester (Zahan et al. 2018).

### Energy assessment

Energy efficiency of the hybrid system consist of MW pretreatment and AD was evaluated considering the energy input (E_i_) for MW heating and energy output (E_0_) in the BMP test. The CH_4_ output was calculated according to the difference between CH_4_ produced in samples and control bottle. Equation 5, 6 were used for the calculation of (E_i_) and (E_0_) (Passos et al. 2014; Kavitha et al. 2018)

5 $$E_{i} = \frac{P \times t}{{g_{VSS} \times V}}$$

6 $${E}_{o}=\Delta C{H}_{4}\times\upxi \times\upeta$$

where: E_i_: (kJ/g VS); P (kW): power; t (hours): heating time; V (L); sample volume; E_0_: (kJ/g VS); ∆YCH_4_ (m^3^ CH_4_/g VS): the CH_4_ yield difference between control and disintegrated samples; ξ: 35,800 kJ/m^3^ CH_4_; η: is assumed to be 90%.

### Analytical method

The sCOD, TS, and VS analyses were performed according to the Standard Methods (APHA 2005). The concentrations of sCOD, NH_4_–N, and PO_4_–P were determined in the clear samples, after centrifugation of supernatant at 4100 rpm (NF 800 NÜVE) and then filtration using 110 mm black bar filter. For the analysis of proteins and carbohydrates, the samples were filtered using Whatman GF/C fine glass filter (pore size: 0.45 µm). Glucose concentrations were extrapolated using a previously established glucose curve. Protein levels were determined using the Biuret method with a Protein Assay Kit (Cat. No. 1.10307.0500). Bovine serum albumin was used as the standard. Merck analytical kits (NH_4_–N: 14752, PO_4_–P: 114848) were used for measuring the concentrations of NH_4_–N and PO_4_–P. While the Anthrone method was applied for the soluble sugars analyze, the soluble proteins were determined with bovine serum albumin as a reference standard. Proteins, sugars, NH_4_–N and PO_4_–P concentrations were measured by using a spectrophotometer (MERCK PHARO 100). The pH measurement was conducted with the Hanna pH meter.

To calculate the theoretical biomethane potential (tBMP, mL CH_4_/g VS), by applying Eq. 7 and 8, the elemental analysis (C, H, O, N, S) was performed at the Scientific and Technological Research Center of Inonu University.

7 $${\text{C}}_{{\text{n}}} {\text{H}}_{{\text{a}}} {\text{O}}_{{\text{b}}} {\text{N}}_{{\text{c}}} + \left( {{\text{n}} - \frac{{\text{a}}}{4} - \frac{{\text{b}}}{2} + \frac{{3{\text{c}}}}{4}} \right){\text{H}}_{2} {\text{O}} \to \left( {\frac{{\text{n}}}{2} + \frac{{\text{a}}}{8} - \frac{{\text{b}}}{4} - \frac{{3{\text{c}}}}{8}} \right){\text{CH}}_{4} + \left( {\frac{{\text{n}}}{2} - \frac{{\text{a}}}{8} + \frac{{\text{b}}}{4} + \frac{{3{\text{c}}}}{8}} \right){\text{CO}}_{2} + {\text{cNH}}_{3}$$

8 $$tBMP(\frac{{mL(CH_{4} )}}{gvs}) = \left( {\frac{{22,4\left( {{n \mathord{\left/ {\vphantom {n {2 + {a \mathord{\left/ {\vphantom {a {8 - {b \mathord{\left/ {\vphantom {b {4 - 3{c \mathord{\left/ {\vphantom {c 8}} \right. \kern-0pt} 8}}}} \right. \kern-0pt} {4 - 3{c \mathord{\left/ {\vphantom {c 8}} \right. \kern-0pt} 8}}}}}} \right. \kern-0pt} {8 - {b \mathord{\left/ {\vphantom {b {4 - 3{c \mathord{\left/ {\vphantom {c 8}} \right. \kern-0pt} 8}}}} \right. \kern-0pt} {4 - 3{c \mathord{\left/ {\vphantom {c 8}} \right. \kern-0pt} 8}}}}}}}} \right. \kern-0pt} {2 + {a \mathord{\left/ {\vphantom {a {8 - {b \mathord{\left/ {\vphantom {b {4 - 3{c \mathord{\left/ {\vphantom {c 8}} \right. \kern-0pt} 8}}}} \right. \kern-0pt} {4 - 3{c \mathord{\left/ {\vphantom {c 8}} \right. \kern-0pt} 8}}}}}} \right. \kern-0pt} {8 - {b \mathord{\left/ {\vphantom {b {4 - 3{c \mathord{\left/ {\vphantom {c 8}} \right. \kern-0pt} 8}}}} \right. \kern-0pt} {4 - 3{c \mathord{\left/ {\vphantom {c 8}} \right. \kern-0pt} 8}}}}}}}} \right) \times 10^{3} }}{(12n + a + 16b + 14c)}} \right)$$

The theoretical oxygen demand (tCOD) of Bs was calculated as mgO_2_/gVS stoichiometrically assuming that the organics was completely degraded to CO_2_, H_2_O and NH_3_ (Eq. 9 and 10).

9 $${C}_{n}{H}_{a}{O}_{b}{N}_{c}+\left(n+\frac{a}{4}-\frac{b}{2}-\frac{3c}{4}\right){O}_{2}\to nC{O}_{2}+\left(\frac{a}{2}-\frac{3c}{8}\right){H}_{2}O+cN{H}_{3}$$

10 $$tCOD = \frac{{\left( {2n + \frac{a}{2} - b - \frac{3c}{2}} \right) \times 16}}{12n + a + 16b + 14c} \times 1000$$

The concentration of free ammonia (NH_3_) (Anthonisen et al. 1976) was calculated by using the Eq. 11, 12, 13 was applied to determine the removal efficiency (E %).

11 $$NH_{3} \left( {mg/L} \right) = \frac{17}{{14}} \times \frac{{\sum NH_{4} - N \left( {mg/L} \right) \times 10^{pH} }}{{e^{{{\raise0.7ex\hbox{${6344}$} \!\mathord{\left/ {\vphantom {{6344} {\left( {273 + T} \right)}}}\right.\kern-0pt} \!\lower0.7ex\hbox{${\left( {273 + T} \right)}$}}}} + 10^{pH} }}$$

12 $$E \left( \% \right) = \frac{{X_{influent} - X_{effluent} }}{{X_{influent} }} \times 10$$

13 $$COD equivalent of consumed VS in the BMP test = \left( {VS_{influent} - VS_{effluent} } \right) \times \frac{gCOD}{{gVS}}$$

where, VS/X_influent_ and VS/X_effluent_: influent and effluent concentrations.

## Results and discussion

### MW studies

The pH of samples was about neutral level. Although a slight decrease of pH was observed with the MW pretreatment, the pH change of samples was negligible (Table 1). Similar pH decrease was observed in our previous study (Alhraishawi et al. 2024). The decrease in pH after MW pretreatment was attributed to the release of hydrogen ions because of disintegration of biosolids (Jackowiak et al. 2011).

**Table 1:** Characteristics of pretreated biosludge before and after centrifugation

Parameter	Unite	Bs	PBs	CBs	PCBs
pH	−	7.17 ± 0.04	6.85 ± 0.08	7.31 ± 0.09	6.89 ± 0.11
TS	g/L	12.7 ± 0.092	10.6 ± 0.251	34.1 ± 0.933	27.9 ± 0.975
VS	g/L	9.2 ± 0.025	8.0 ± 0.19	24.6 ± 1.633	21.1 ± 1.475
sCOD	mg/L	72.6 ± 6	2986 ± 126	81.4 ± 11	6400 ± 320
Protein	g/L	0.385 ± 0.025	1.08 ± 0.019	0.42 ± 0.04	2.16 ± 0.15
Sugar	mg/L	7.5 ± 0.55	78 ± 3	10.4 ± 1.6	216.4 ± 6.6
NH_4_ − N	mg/L	3.6 ± 0.02	11.5 ± 2	4.8 ± 0.03	22 ± 1.6
PO_4_ − P	mg/L	6.6 ± 0.4	23 ± 1.4	8.4 ± 2.1	38 ± 3.5
Specific energy consumption	kJ/gVS	−	301	−	108.8

#### Variations of solids concentration

The volatile organics is generally considered as a fundamental parameter to evaluate the hydrolysis of Bs with the pretreatment methods (Fang et al. 2017). The initial TS and VS concentrations of Bs were 12.7 g/L and 9.2 g/L, and after centrifugation, increased to 34.1 g/L and 24.6 g/L, respectively. The pretreatment of samples caused an average decrease of 17% and 14% in TS and VS, respectively. MW irradiation was successfully destroyed the Bs structure and organics were released into the supernatant. By the TS content reduction with the MW heating, the decrease of Bs was reported by Rani et al. (2017). It was reported in the literature that the decrease of TS/SS/VS concentrations in Bs by MW irradiation were varied between about 10% and 31% depended on the Bs composition, irradiation temperature, MW intensity, and time (Saha et al. 2011; Rani et al. 2013; Hephzibah et al. 2015; Fang et al. 2017). A higher solubilization of solids by pretreatment cause the elimination of hydrolysis step of AD process (Rani et al. 2013).

#### Release of organic substances

Negligible difference between the sCOD concentrations in Bs of about 72 mg/L and CBs 81 mg/L was observed (Table 1). With the MW irradiation significantly increase the release of organic contents and the sCOD concentrations of about 3000 mg/L and 6400 mg/L were determined in the PBs and PCBs, respectively. Although the difference of initial sCOD concentration of Bs and CBs was too low, elevation of TS concentration, about two times higher sCOD release in the PCBs was obtained compared to the PBs by the MW irradiation.

Significant increase in the organic contents by thermal pretreatment of Bs containing high TS was reported (Fang et al. 2017; Jeong et al. 2019; Jiang et al. 2021). Gil et al. (2019) reported that the sCOD release increased significantly in the CBs pretreated at a specific energy level of 18,000 kJ/kg TS. It was determined that the raw Bs solubility (sCOD/tCOD) of 6% rose to 16 − 17% in the CBs, which was subjected to the MW power of 400 W and temperatures ranging between 90 °C and 120 °C (Park et al. 2010). Due to high viscosity, which restricted the heat transfer in Bss with very high TS content, a deterioration in solubility and corresponding decrease in sCOD concentration was observed (Ahn et al. 2021).

Both intracellular and extracellular biopolymers such as proteins, sugars, lipids and nucleic acids constitute the organic part of Bs. Soluble biopolymers released after pretreatment are considered as a good indicator for evaluating the solubility of organic compounds in Bs (Eskicioglu et al. 2007a). The pretreatment process might led to increase the release of proteins and polysaccharides into the soluble fraction of EPS was reported by Liu et al. (2016). The released solubilization of biopolymers into the supernatant with the MW pretreatment of Bs, was monitored by protein and sugar in this study.

The protein concentration was 0.385 g/L and 1.08 g/L in Bs and PBs, respectively. While the content in CBs was 0.42 g/L, protein concentration increased to about 2.2 g/L after the MW pretreatment. A significant improvement in protein concentration of PCBs was determined which was about two times higher than that of PBs. As observed from the release of sCOD and protein, the sugar content of Bs, about 10 mg/L, was improved about 10 and 22 times for PBs and PCBs, respectively, after the MW disintegration. With the lack of caramelization and high sugar content in liquor indicated that the MW operating conditions were suitable for biosolid degradation. However, it could be concluded from the increase of protein content, the MW irradiation was not effective for protein degradation.

Previous experimental studies were also indicated the release of organics from CBs improved significantly after MW pretreatment at various operational conditions (Yan et al. 2013; Mehdizadeh et al. 2013; Liu et al. 2016; Kor − Bicakci et al. 2019).

The specific energy consumption for MW irradiation for the PBs and PCBs was 301 kJ/gVS and about 109 kJ/gVS, respectively. Increase of Bs TS content declined the MW energy requirement of about 3 times. Tang et al. (2010) observed that high water content prevented the biosolids from dissolving at given energy, but low water level might promote the breakdown of organic matter with a lower energy input. Jeong et al. (2019) reported that it was necessary to increase the energy input or extend the residence time in thermal pretreatment of Bs with solids concentration higher than 10%.

#### Release of nutrients

The initial NH_4_ − N concentration was 3.6 mg/L and 4.8 mg/L for Bs and CBs, respectively. By the pretreatment, significantly increased to about 12 mg NH_4_ − N/L and 22 mg NH_4_ − N/L, for PBs and CPBs respectively. Similar elevation was also observed for PO_4_ − P after MW irradiation. The highest PO_4_ − P concentration of approximately 38 mg/L was determined in the PCBs.

The concentrations of NH_4_ − N and PO_4_ − P in the pretreated samples in this experiment were within the range reported in previous studies under different MW operating conditions (Aslan et al. 2024). Low NH_4_ − N and PO_4_ − P concentrations in the liquor after thermal pretreatment were attributed to the NH_3_ evaporation (Eskicioglu et al. 2008) and precipitation of phosphate or formation of complexation of PO^2−^_4_ (Kataki et al. 2016) at high temperatures, respectively. The biosolids contain high amounts of P and N, mainly in the structure of proteins. As could be seen from the NH_4_ − N concentration in the disintegrated Bs, the MW irradiation was not effective in the N release from the protein contain about 16 − 19% N (Suschka and Grübel 2014).

The MW process led to more than twofold increase in NH_4_ − N concentration release from the Bs. However, NH_4_ − N and protein concentrations in the pretreated samples indicated that the MW process was not effective on the protein degradation.

### BMP assay

#### sCOD variation

Prior to the BMP test, the sCOD concentrations of disintegrated Bs namely CBs and PCBs were significantly greater than the control tests fed with the Bs. The sCOD concentrations at the beginning and end of BMP tests, which were conducted throughout 71 days, are presented in Table 2. Initial sCOD concentration was about 286 mg/L in the control BMP bottle, while decreased to 209 mg/L at the end of test. Initial sCOD concentration of CBs fed to BMP unit were very close to Bs, while increase to 498 mg/L was determined at the end of test. The sCOD concentration in the effluents were high which was consistent with the study of Liu et al. (2017). Compared to Bs and CBs substrates, the initial sCOD concentrations were quite high about 1700 mg/L and 5800 mg/L for the PBs and PCBs fed bottles, respectively. After the AD, the sCOD levels decreased to 486 mg/L and 1550 mg/L for the PBs and PCBs substrates, respectively.

**Table 2:** Characteristics of wastewaters produced in the BMP test

Parameter	Unite	Bs		PBs		CBs		PCBs	
		Initial	Final	Initial	Final	Initial	Final	Initial	Final
pH	−	6.87 ± 0.02	8.15 ± 0.01	7.03 ± 0.04	8.11 ± 0.02	6.91 ± 0.03	8.81 ± 0.01	7.01 ± 0.03	8.90 ± 0.02
TS	g/L	11.1 ± 0.2	9.3 ± 0.1	11.2 ± 0.2	8.7 ± 0.2	23.1 ± 0.7	18.2 ± 0.5	23.2 ± 0.5	16.7 ± 0.4
VS	g/L	7.7 ± 0.2	5.7 ± 0.2	7.7 ± 0.1	5.2 ± 0.1	16.9 ± 0.8	11.7 ± 0.3	17.1 ± 0.6	10.3 ± 0.3
sCOD	mg/L	286 ± 31	209 ± 8.5	1,700 ± 80	486 ± 76	295 ± 22	498 ± 46	5,780 ± 333	1,550 ± 88
Protein	g/L	0.328 ± 0.003	0.1355 ± 0.02	0.56 ± 0.01	0.166 ± 0.01	0.3075 ± 0.01	0.088 ± 0.02	0.744 ± 0.02	0.081 ± 0
Sugar	mg/L	9.4 ± 0.6	2.5 ± 0.14	42.3 ± 2.3	6.01 ± 0.74	10.3 ± 1.7	3.7 ± 0.4	89 ± 7	13.3 ± 0.3
NH_4_ − N	mg/L	85 ± 3.25	284 ± 12	87 ± 5	339 ± 9	84 ± 11.5	702 ± 15	93 ± 8	717 ± 18.3
PO_4_ − P	mg/L	62 ± 3.25	83 ± 4.2	67 ± 4.4	94 ± 2.5	63 ± 7.1	205 ± 6.8	76.3 ± 5.1	215 ± 10

Although the TS content of CBs was doubled compared to the Bs, the initial sCOD concentrations were very close. The organic fraction released by the breakdown of biomass during AD process was consumed by bacteria and the fraction that was difficult to degrade and/or non − biodegradable remained in the liquid at the end of test. The increase in sCOD concentration in effluent of BMP fed with CBs was due to the high TS content. During the anaerobic degradation process, it was observed an upward trend of sCOD when the Bs containing 9–10% solids was fed to the reactor due to the accumulation of intermediate products such as volatile fatty acid (VFA). Two main disadvantages of pretreatment before the AD were the high sCOD and NH_3_ concentrations in the digested effluent supernatant (Eskicioglu et al. 2006). Liu et al. (2017) reported that the non − biodegradable sCOD concentrations, which could not be utilized by bacteria in AD process, in the supernatant increased with the pretreatment.

The sCOD decrease in the effluent of BMP units fed with the pretreated Bss indicated that the MW pretreatment process was effective in producing biodegradable organic matter in the AD process. Additionally, the decrease in TS and VS concentrations at the end of AD process proved the organic compounds were effectively consumed by the bacteria. Saha et al. (2011) reported that the thermal pretreatment of Bs at 90 °C could improve the sCOD reduction because of the partial decomposition or disruption of biosolid structure.

#### Protein and sugar variations

As can be seen from the Table 2 that by the utilization of protein, concentrations decreased notably in all units. It was determined that the decrease of protein concentrations in the BMP units fed with pretreated Bs were significant compared to the raw Bs. The removal efficiency at BMP units fed with the Bs and PBs were about 59 and 71%, respectively. While the protein consumption ratio in the BMP units containing CBs was about 74% and increased to 89% for the PCBs. Compared to the study conducted by Houtmeyers et al. (2014), which was determined the removal efficiency of about 31 and 11% for the MW disintegrated Bs and control, respectively, significantly higher protein consumption was observed in this study.

The sugars removal efficiency was greater in the pretreated reactors than in the control group, as was also the case with proteins. In the bottles fed with Bs, the decrease rate of sugar was about 61% while it increased to 86% in the PBs added unit. It was reported that after the disintegration of Bs by MW irradiation, the removal efficiency of protein and carbohydrates in the AD process was improved (Zou et al. 2020). Zou et al. (2020) indicated that the soluble organic matter in the liquid increased significantly after MW pretreatment and in the subsequent AD reaction, bacteria rapidly utilized released organic compounds, including proteins and sugars compared to the control unit.

#### NH_4_ − N and PO_4_ − P variations

Initially, the concentrations of NH_4_ − N in units contained Bs and PBs were about 86 mg/L, while after the AD, they increased significantly to about 284 mg/L and 340 mg/L, respectively. While the initial NH_4_ − N concentration was lower than about 95 mg/L in CBs and PCBs, it increased to about 710 mg/L in the supernatant. Compared to bottles fed with raw Bs and CBs, NH_4_ − N release was observed to be slightly higher in pretreated samples (PBs and PCBs), which was consistent with the study conducted by Zou et al. (2020). Throughout the AD process, because the complex organic molecules, including proteins, were broken down into simpler compounds such as ammonia, increase of NH_4_ − N concentration in the effluent was observed (Zhang et al. 2017).

One of the most important inhibitor of AD process, especially for methanogens, is unionized ammonia (NH_3_)/free ammonia (FA), which across the cell membrane of organisms. The increase in FA concentration in digester shift the FA to one of other form of nitrogen compounds namely ionized ammonia (NH^+^_4_) that causes an increase in pH and also more toxicity for organisms (Zahan et al. 2018).

By the degradation of proteins during the AD process, the activity of anaerobic bacteria may be inhibited if NH_3_ concentration in the reactor exceeds the inhibition threshold concentration, depending on the temperature and pH levels. The calculated NH_3_ concentration in the BMP units were between 14 mg/L and 34 mg/L. The threshold concentration of NH_3_ − N was reported as 55 ± 11 mg/L (Bhattacharya and Parkin 1989) and 80 mg/L (Braun et al. 1981) for the AD process. According to the BMP test conditions performed at 35 ± 2 °C temperature and 7.5 ± 0.5 pH values, respectively, bacteria was not affected since the NH_3_ concentration was lower than the threshold level.

Although high amounts of NH_4_ − N were released, especially in BMP units fed with centrifuged substrates, no inhibition effect on the AD process was observed. Liao et al. (2016) also did not observe any biological reaction limitation at high TN (NH_4_ + NH_3_) concentrations reaching up to 2,845 mg/L in the reactor.

As observed in the release of NH_4_ − N after AD process, the concentration of PO_4_ − P also increased from an average value of 63 mg/L in controls to about 83 mg/L and 94 mg/L in BMPs fed with Bs and PBs, respectively. The initial concentrations of PO_4_ − P were lower than 80 mg/L before the BMP test, while they increased significantly in the BMP bottles contained PCBs and CBs to about 215 mg PO_4_ − P/L.

#### TS and VS variations

The TS and VS concentrations in supernatants obtained after BMP test were lower than the initial levels. In the Bs fed bottles, the TS decrease of 16.5% was determined, on the other hand the decline of VS was about 26%. In the BMP unit containing PBs, the reduction of TS and VS was about 23 and 32%, respectively. As expected, the decrease in TS and VS in bottles containing higher concentrations of biomass was higher than in those containing lower concentrations. The rate of TS and VS reduction in the CBs was 21 and 31%, while in the PCBs containing bottles was reached to 29 and 40%, respectively. Kor − Bicakci et al. (2019) determined about 10% higher VS reduction in the centrifuged Bs, which was pretreated with MW irradiation compared to the control reactor. The higher VS reduction in AD process indicated the more effective consumption of organic compounds. Di Capua et al. (2020) reported that the pretreatment of Bs with high TS content improved the hydrolysis rate, VS conversion efficiency and consequently biomethane production in AD. Liu et al. (2018) also reported VS reduction of about 35% and 47% in the centrifuged Bs for control and MW pretreated Bs after 60 days AD process, respectively.

#### CH_4_ production

The cumulative CH_4_ production in BMP units fed with Bs and PBs were 368 mL and 461 mg/L, respectively. In the bottles contained CBs, the cumulative CH_4_ production was about 903 mL, and the highest CH_4_ volume of 1126 mL was obtained by feeding PCBs with the improvement of about 25% compared to control.

**Fig. 1:**
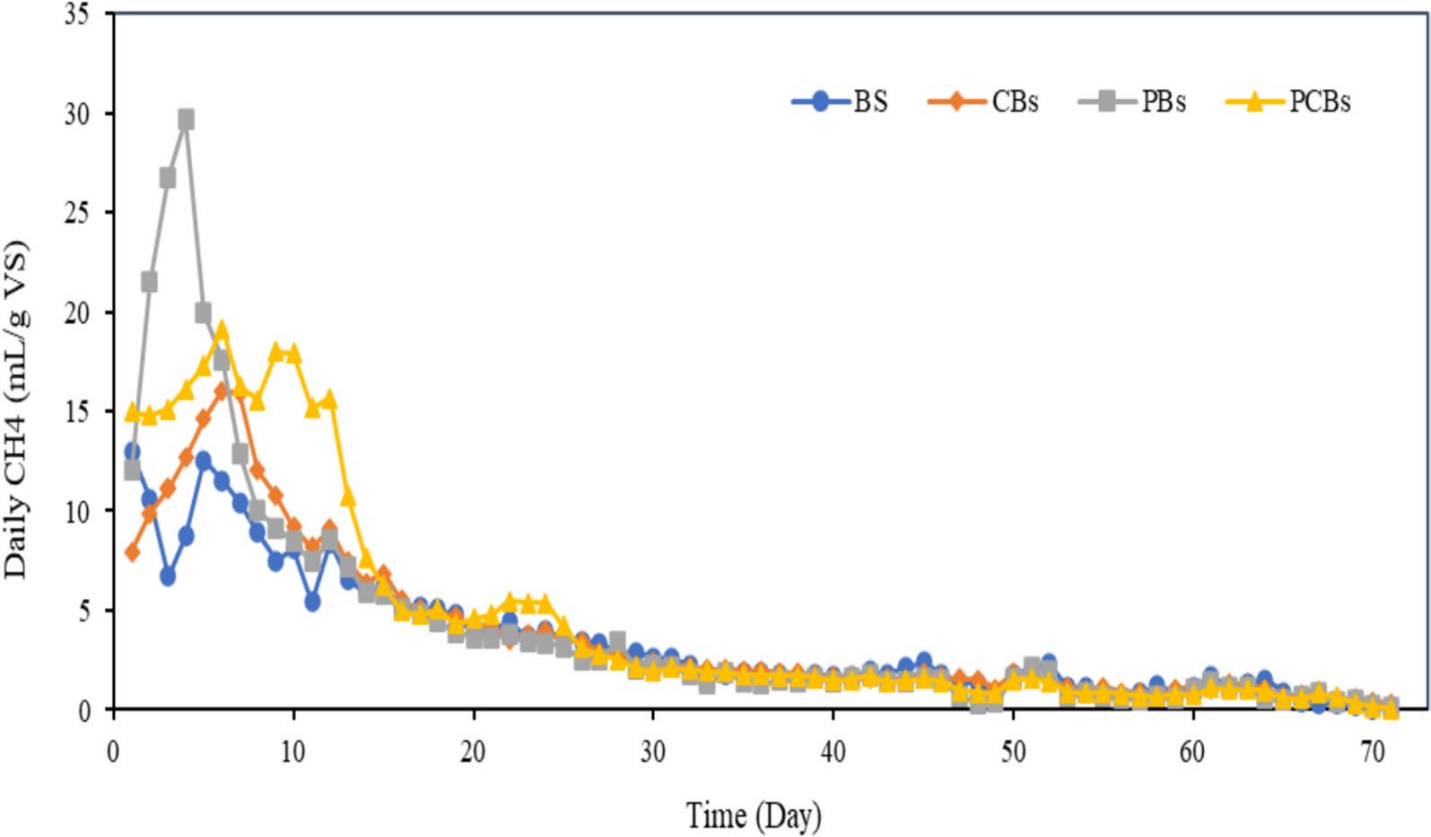
Daily CH_4_ production

**Fig. 2:**
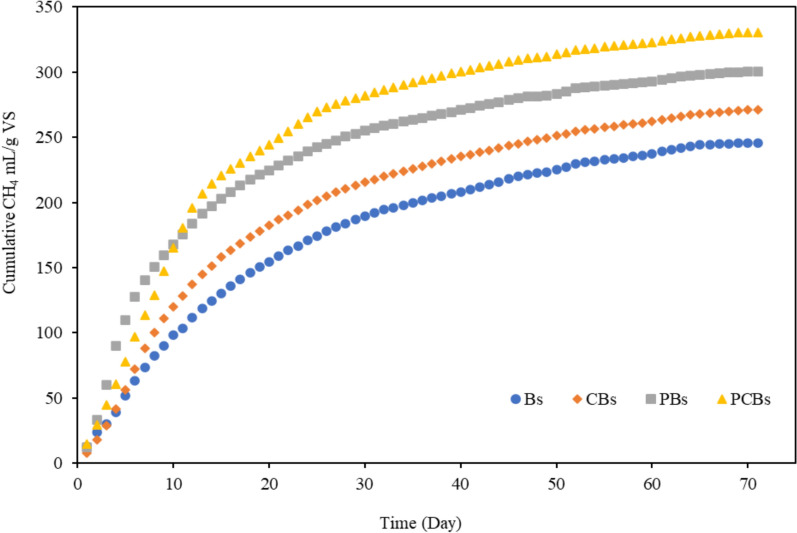
Cumulative CH_4_ production

The daily and cumulative CH_4_ production by feeding the pretreated and raw Bs are depicted in Figs. 1 and 2, respectively. Compared to the controls without pretreatment, higher CH_4_ production were observed in the BMP units contained pretreated Bs. The highest daily CH_4_ production rates were 12.5 mL/g VS.day with the Bs and 29.6 mL/g VS.day in unit fed with the PBs. The highest daily CH_4_ production rates of 16 mL/gVS.day and 19 mL/g VS.day were obtained for the CBs and PCBs, respectively.

At the end of first week, CH_4_ gas production started to decrease across all BMP units due to the rapid consumption of easily biodegradable organic substrates. However, the decrease rate of CH_4_ production in PCBs was lower compared to the others, because of the positive effect of pretreatment of Bs, which released the organics more abundantly with high TS content. This resulted in prolonged CH_4_ production in units with high TS content compared to other reactors. Kavitha et al. (2018) also reported during the initial five days of CH_4_ generation with a rapid increase in production. However, Azizi et al. (2019) observed low CH_4_ production in the first days of operation in AD process fed with pretreated domestic sludge cake at 160 °C with decrease in sugar and protein levels as a result of Maillard reaction.

Compared with untreated Bs, the reason for improved CH_4_ production from pretreated Bs was the higher solubility and digestibility of Bs. In addition, Hephzibah et al. (2015) reported that a high CH_4_ volume production could be achieved when the MW intensity was increased at a slower heating rate.

In the last days of experiments, there was little variation in CH_4_ production between BMP units, which could be attributed to prove sufficient time for the disintegration of hard to degrade organic compounds, which might be formed by the MW pretreatment (Kor − Bicakci et al. 2019) and Bs with high TS content (Liao et al. 2014).

The observed cumulative CH_4_ yield was about 300 mL/gVS and 330 mL/gVS for the PBs and PCBs, respectively, while they were about 246 mL/g VS and 271 mL/g VS for the Bs and CBs. The cumulative CH_4_ production was about 22% higher for both the PBs and PCBs fed reactors than the control units.

The MW pretreatment conditions and organic and TS/VS constituents of Bs effect the yield of CH_4_ production in the AD process. Feeding of high concentrations of Bs into the AD reactor, the biodegradation rates could be inclined due to the restriction of microbial activity or the limitation of essential nutrients and enzymes transportation (Liao et al. 2016). After the MW pretreatment of Bs with a solid content of 5.4% at 96 °C, 21% increase in CH_4_ production was achieved compared to the control, while at a lower solid content of 1.3%, increase in gas production of about 15% was observed (Eskicioglu et al. 2007b). Yan et al. (2013) observed that the dewatered and pretreated biomass at the temperatures ranging between 90  and  100 °C for a duration pretreatment of 30 min provided in the biogas yields ranging from about 85 to 143 mL/g VS, compared to a control group 18.3 mL/g VS. With a high solid content (8 − 8.5%) of Bs was disintegrated in the MW at 100 °C and 600 W, 25% increase in CH_4_ production was determined compared to the control group (Liu et al. 2018).

The pretreatment of Bs at the moderate MW temperatures ( < 100 °C) and convenient irradiation time/heating rates could enhance CH_4_ production without inhibiting the following AD process, whilst it could be restricted at high temperatures (Azizi et al. 2019). It was reported that the concentrated Bs pretreated at 80 °C for duration of 90 min resulted in the specific CH_4_ production increased to 303 mL of CH_4_/g VS, while the biodegradability elevated to 73% (Veluchamy and Kalamdhad 2017). Yan et al. (2013) observed that the dewatered and pretreated biomass at the temperatures ranging between 90 − 100 °C for duration pretreatment of 30 min provided in the CH_4_ yields in the AD reactor ranging from about 85 mL/g VS to 143 mL/g VS, compared to a control group 18.3 mL/g VS.

It could be concluded that the CH_4_ yield in AD process could be significantly increased by the pretreated Bs with MW and increasing the TS content. In order to ensure homogeneous Bs mixing in the operation of AD reactor, the TS concentration should be optimized. MW operating conditions must be also optimized in order to achieve the highest CH_4_ production possible, taking into account energy consumption in the MW operation.

The tBMP could be calculated considering the biosludge elemental compositions (Alhraishawi et al. 2024 and 2025) or organic fractions such as lipids, proteins and carbohydrates (Paletta et al. 2023). Based on the stoichiometric composition of Bs, the empirical formula was defined as C_6.4_H_11.4_O_2.6_N. From the elemental composition by using Eq. 7, the tBMP for Bs was calculated as 560 mL of CH_4_/g VS. The biodegradability of Bs and CBs was 44 and 51%, respectively, after the MW pretreatment, it elevated to about 54% and 59% for PBs and PCBs, respectively.

By using the basic chemical formula of organisms, the tCOD of Bs was calculated about 1.6 g O_2_/g VS. Considering the consumed VS by applying Eq. 12 throughout the BMP test and the COD concentrations at the initial and end of the test, more than 90% COD removal efficiencies were determined in the BMP tests.

It was found that the biodegradability of Bs contained high concentration of TS was higher than the raw Bs, which was contradict with the results of conducted by Yuan et al. (2019). Yuan et al. (2019) explained this reason of results because of the dewatering of Bs was carried out with the use of high molecular weight polyacrylamide − based flocculants as drying agent, high concentrations of Bs about 20%, which negatively affect the biodegradation rates by hindering the microbial activity or transport of essential nutrients and enzymes (Liao et al. 2016).

#### Energy assessment

The produced energy in BMP tests for both PBs and CBs were obtained lower than the energy requirement of MW irradiation under the operational conditions. Compared with the MW energy input of PBs, the E_i_ value of PCBs was about 3 times lower due to the higher VS concentration (Table 3). One important finding in this study was the E_o_/E_i_ ratio for the CBs was also less than 1, indicating a low energy output considering the input. However, because of lower energy input of about 64%, significant increase of E_o_/E_i_ ratio from 0.0058 to about 0.017 was determined by feeding the AD process with the CBs.

**Table 3:** Energy analysis of biosludges with different properties after AD

TS content %	E_i_ kJ/gVS	Reactor code	Ultimate CH_4_ (mL/g VS)	^a^∆CH_4_ (m^3^/g VS)	E_o_ (kJ/gVS)	E_o_/E_i_
1.27	301	Bs	245.4	5.49 × 10^−5^	1.76	0.0058
		PBs	300.3	5.9 × 10^−5^	1.90	0.017
3.4	108.4	CBs	271.4			
		PCBs	330.4			

a: represents the result of energy output after applying the CH_4_ production difference

It was reported that the energy consumption for Bs was found to be about 412 kJ/g TS, while it decreased by 4 times to 104 kJ/g TS for 10% TS (Saha et al. 2011). Kor − Bicakci et al. (2019) observed that the reduction of energy consumption between 54 and 94% by the elevation of solids content in Bs, prior to the pretreatment with MW and UV.

The CH_4_ production capacity of PBs and PCBs was 283 and 377 kJ/gVS_utilized_, respectively, while the Bs and CBs was 203 and 242 kJ/gVS_utilized_. The CH_4_ production yield could be significantly improved by about 32% with the centrifugation of Bs, while it was determined as approximately 36% with the pretreatment of CBs, and the increase was only about 4%.

Liu et al. (2016) reported that the MW disintegration of Bs with solids concentration higher than 3% could increase the CH_4_ production efficiency in AD process and lead to self-sustainable energy production or even produced excess energy. Although the increase in CH_4_ yield was detected in AD process by centrifuging Bs before the MW disintegration, the increase was not sufficient to consider the process was economic under operating conditions in this study. Mehdizadeh et al. (2013) stated that the volume requirement of the anaerobic digester would be lower for thermally pretreated domestic sludge. In the economic evaluation of hybrid system, it should also be taken into account that the anaerobic digester volume will be even lowered with the pretreatment of CBs, compared to the raw Bs.

#### Kinetic studies

**Fig. 3:**
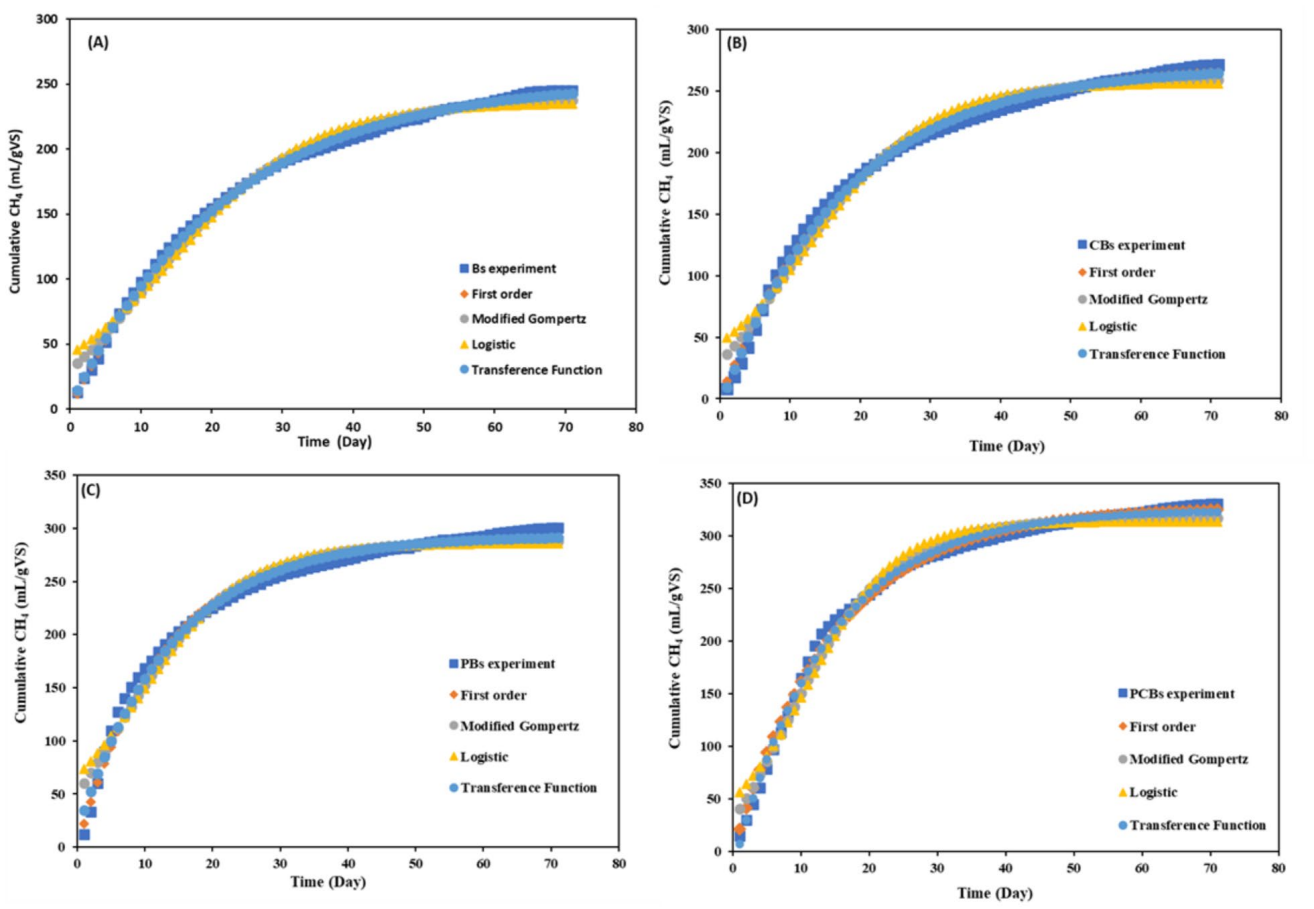
Predict of cumulative CH_4_ production for Bs reactor (**A**), CBs reactor (**B**), PBs reactor (**C**) and PCBs reactor (**D**)

CH_4_ production data from the AD of both raw and pretreated Bs were analyzed using four kinetic models to estimate CH_4_ yield and associated kinetic parameters (Fig. 3, Table 4). As can be seen in figures that the simulated results closely aligned with the experimental data. Although the R^2^ of all models applied were greater than 0.95, the Transference Function and First − Order models had the highest R^2^ values ranging between 0.988 and 0.998. The results is consistent with previous studies (Ugwu and Enweremadu 2019; Aslan et al. 2024).

**Table 4:** The calculated kinetic parameters of models

Model	Reactor	R^2^	A_0_ (mL/gVS)	µ_m_ (mL/gVS.day)	K (d^−1^)	λ (day)
First order	Bs	0.998	251.5	−	0.047	−
	CBs	0.995	271		0.055	
	PBs	0.986	290.6		0.079	
	PCBs	0.995	324.7		0.075	
Gompertz modified	Bs	0.988	240.5	6.6	−	− 3.6
	CBs	0.981	260.4	8.4		− 2.66
	PBs	0.967	288.3	10.6		− 3.11
	PCBs	0.984	317	13.2		− 1.4
Logistic	Bs	0.97	235.7	6		− 4.7
	CBs	0.966	256	7.5		− 3.44
	PBs	0.954	285.8	9.13		− 6.3
	PCBs	0.97	313.6	12		− 1.26
Transference Function	Bs	0.998	252.7	11.5		− 0.293
	CBs	0.998	269.6	15.2		0.357
	PBs	0.988	294.9	21.2		− 0.71
	PCBs	0.993	328.8	32.6		0.681
Experiment	Bs	0.976	−	12.9	0.048	0
	CBs	0.984		16	0.044	0
	PBs	0.991		29.6	0.053	0
	PCBs	0.972		19	0.055	0

The production potential and maximum production rate of CH_4_, hydrolysis rate, and lag phase were estimated by interpreting kinetic model parameters that best fit the experimental results. As can be seen from the Table 4 that the k value obtained from the First − Order model, increased by the disintegration of Bs compared to the control. The high k value indicated improved bioavailability and high rate of the AD process (Ugwu and Enweremadu 2019), while λ values lower than zero prove that the CH_4_ production started immediately (Khadka et al. 2022). Conversely, by the Transference Function model, the highest λ values, which suggested that the bacteria required longer time for utilizing the substrates, were determined for the CBs and PCBs. Results are consistent with a study by Akbay (2024), which reported that the expected lag phase at the Transference Function model for all organic compounds was less than one day. In the Transference Function model, the highest A_0_ values for PBs and PCBs were determined to be approximately 295 mL/g VS and 329 mL/g VS, while lower values of approximately 253 mL/g VS and 270 mL/g VS were observed for the Bs and CBs samples, respectively. The highest μ_m_ values, which were notably higher compared to other models, were observed for the Transference Function model. The μ_m_ value for the Bs and CBs were about 11.5 mL/gVS.day and 15.2 mL/gVS.day, respectively, while it was determined that approximately two times higher for the PBs and PCBs. The findings are consistent with previous studies showing that the Transference Function and First − Order models have higher A_0_ (Ugwu and Enweremadu 2019) and µ_m_ (Li et al. 2012) values compared to Logistic and modified Gompertz models. Syaichurrozi et al. (2020) reported that the μ_m_ value increased with the A_0_ value in theoretically, which implies that the maximum CH_4_ potential yield would form at a higher rate of CH_4_ production. The results showed significant increase in potential and maximum daily production rate of CH_4_ production with the pretreated Bs.

The cumulative CH_4_ volumes difference between the obtained experimentally and predicted by First order and Transference Function models were between 1.2 − 3.5% and 0.79 − 2.9%, respectively. Whilst the differences for modified Gompertz and Logistic models were higher than the others, it was determined that was lower than 5.5%. Although the Transference Function was the model with the lowest deviation value, the models with deviation rates lower than 10% were considered valid in the literature (Ugwu and Enweremadu 2019; Syaichurrozi et al. 2020).

## Conclusion

In this study, the energy efficiency of hybrid system consist of sequential MW and AD processes was investigated for the raw Bs and CBs. By the MW irradiation, TS and VS concentrations in the liquor was declined due to the breakdown of organisms. The MW irradiation significantly improved the release of organic compounds from the biosolids and leading to the increase of 23% and 21% in CH_4_ production for pretreated Bs and CBs, respectively, compared to controls. After the AD process significant amount of NH_4_ − N and PO_4_ − P were released into the supernatant. However, the MW irradiation was not effective on the nutrient release especially NH_4_ − N because of the ineffective protein degradation in the MW heating. Although the applied kinetic models provided well fit with the experimental data, the highest R^2^ values determined for the Transference Function and First − Order models.

Although energy production in the hybrid system fed with CBs did not appear to be an economical when the process was evaluated only in terms of energy efficiency, it was found that the energy consumption was about three times lower than the unit fed with raw Bs. However, not only the MW energy consumption but also the reduction in AD reactor volume and the increase in CH_4_ production should be taken into account when evaluating the hybrid system economically.

## Data Availability

All data generated or analyzed during the study were presented in article.

## Abbreviations

AD: Anaerobic digestion
BMP: Biochemical methane potential
Bs: Biosludge
E_i_: Energy input
E_o_: Energy output
CBs: Centrifuged Bs
HRT: Hydraulic retention time
PBs: Pretreated Bs
PCBs: Pretreated centrifuged Bs
Mw: Microwave
sCOD: Soluble chemical oxygen demand
tBMP: Theoretical BMP
TS: Total solid
VS: Volatile solid
WWTP: Wastewater treatment plant
